# Regulation of formin INF2 and its alteration in INF2-linked inherited disorders

**DOI:** 10.1007/s00018-024-05499-3

**Published:** 2024-11-25

**Authors:** Leticia Labat-de-Hoz, M. Ángeles Jiménez, Isabel Correas, Miguel A. Alonso

**Affiliations:** 1 Centro de Biología Molecular Severo Ochoa (CBMSO), Consejo Superior de Investigaciones Científicas (CSIC), Universidad Autónoma de Madrid (UAM), 28049 Madrid, Spain; 2 Instituto de Química Física (IQF) Blas Cabrera, Consejo Superior de Investigaciones Científicas, 28006 Madrid, Spain; 3 Department of Molecular Biology, UAM, 28049 Madrid, Spain

**Keywords:** Focal segmental glomerulosclerosis, Charcot–Marie–Tooth disease, Pathogenic variants, Podocytes, Mitotic catastrophe, Actin

## Abstract

Formins are proteins that catalyze the formation of linear filaments made of actin. INF2, a formin, is crucial for correct vesicular transport, microtubule stability and mitochondrial division. Its activity is regulated by a complex of cyclase-associated protein and lysine-acetylated G-actin (KAc-actin), which helps INF2 adopt an inactive conformation through the association of its N-terminal diaphanous inhibitory domain (DID) with its C-terminal diaphanous autoinhibitory domain. INF2 activation can occur through calmodulin binding, KAc-actin deacetylation, G-actin binding, or association with the Cdc42 GTPase. Mutations in the INF2 DID are linked to focal segmental glomerulosclerosis (FSGS), affecting podocytes, and Charcot-Marie-Tooth disease, which affects Schwann cells and leads to axonal loss. At least 80 pathogenic DID variants of INF2 have been identified, with potential for many more. These mutations disrupt INF2 regulation, leading to excessive actin polymerization. This in turn causes altered intracellular trafficking, abnormal mitochondrial dynamics, and profound transcriptional reprogramming via the MRTF/SRF complex, resulting in mitotic abnormalities and p53-mediated cell death. This sequence of events could be responsible for progressive podocyte loss during glomerular degeneration in FSGS patients. Pharmacological targeting of INF2 or actin polymerization could offer the therapeutic potential to halt the progression of FSGS and improve outcomes for patients with INF2-linked disease.

**Supplementary Information:**

The online version contains supplementary material available at 10.1007/s00018-024-05499-3.

## The formins

**Fig. 1:**
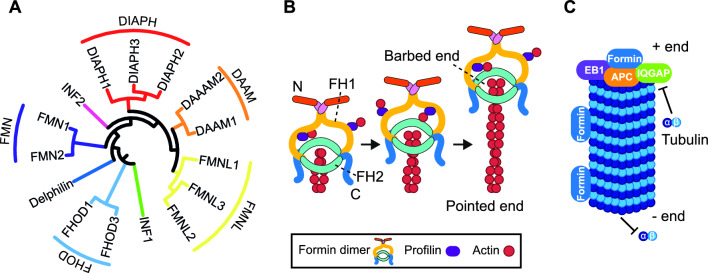
The formin family and functions. **A** Tree of human formins. The tree was constructed using the Muscle algorithm of Mega software (megasoftware.net/home; version 10.1.8), aligning the FH2 sequences of the proteins. The Uniprot protein accession numbers of the corresponding sequences are DIAPH1 (O60610), DIAPH2 (O60879), DIAPH3 (Q9NSV4), DAAM1 (Q9Y4D1), DAAM2 (Q86T65), FMNL1 (O95466), FMNL2 (Q96PY5), FMNL3 (Q8IVF7), FHOD1 (Q9Y613), FHOD3 (Q2V2M9), INF2 (Q27J81), FMN1 (Q68DA7), FMN2 (Q9NZ56), Delphilin (A4D2P6) and INF1 (Q9C0D6). **B** Actin polymerization by formins. The FH2 domains of a formin dimer nucleate the formation of an actin polymer, assisted by profilin and the formin C-terminal segment. The formin remains bound to the barbed end during elongation. **C** Model of microtubule stabilization. Formins interact with other proteins, such as the microtubule plus-end tracking proteins EB1 and APC, and the scaffolding protein IQGAP1, to form a complex that caps the growing + end of microtubules and inhibits the addition of new tubulin subunits. In addition, formins bind laterally along microtubules via the FH2 domain, contributing to the stabilization and protection of microtubules against disassembly. APC, adenomatous polyposis coli; EB1, end-binding protein 1; FH1, formin homology 1 domain; FH2, formin homology 2 domain; IQGAP, IQ motif-containing GTPase-activating protein

Formins are a family of proteins comprising fifteen members in humans, some of which have isoforms resulting from alternative splicing (Fig. 1A) [1]. These proteins catalyze the polymerization of monomeric globular actin (G-actin) into linear filamentous actin (F-actin) [2]. A defining feature of formins is their core region, which includes a formin homology (FH) 2 domain that is responsible for actin nucleation. This domain is preceded by a proline-rich FH1 domain that recruits the G-actin-binding protein profilin. The process of actin polymerization by formins involves a multistep mechanism by which the FH2 domain of a formin dimer stabilizes an actin dimer and, with the aid of profilin-bound G-actin, elongates the actin filament. Throughout this process, the FH2 dimer remains attached to the growing (“barbed”) end of the filament, facilitating the addition of more actin subunits (Fig. 1B) [3].

In addition to their crucial role in building the actin cytoskeleton, formins interact with microtubules, influencing their stability, posttranslational modifications, and alignment with actin stress fibers [2]. To stabilize microtubules, formins assemble a protein complex at the growing (“ + ”) end, making them refractory to further elongation, and bind along the lateral side to protect the microtubules from disassembly (Fig. 1C) [4, 5].

Formins are integral to numerous cellular processes, such as mitosis, cytokinesis, cell adhesion and migration, cell polarity, membrane trafficking, and cell and tissue morphogenesis [2]. Given their essential functions, it is unsurprising that alterations in formins can lead to disease. Mutations in seven formin genes (DIAPH1-3, DAAM2, FMN2, FHOD3 and INF2) are the genetic basis of various inherited human disorders, such as intellectual disability, renal disease, peripheral neuropathy, thrombocytopenia, primary ovarian insufficiency, hearing loss and cardiomyopathy. Additionally, mutation or dysregulation of these and other formins is associated with a range of pathological conditions, including developmental defects, aging-related diseases and cancer [6].

## Structure and regulation of formins

**Fig. 2:**
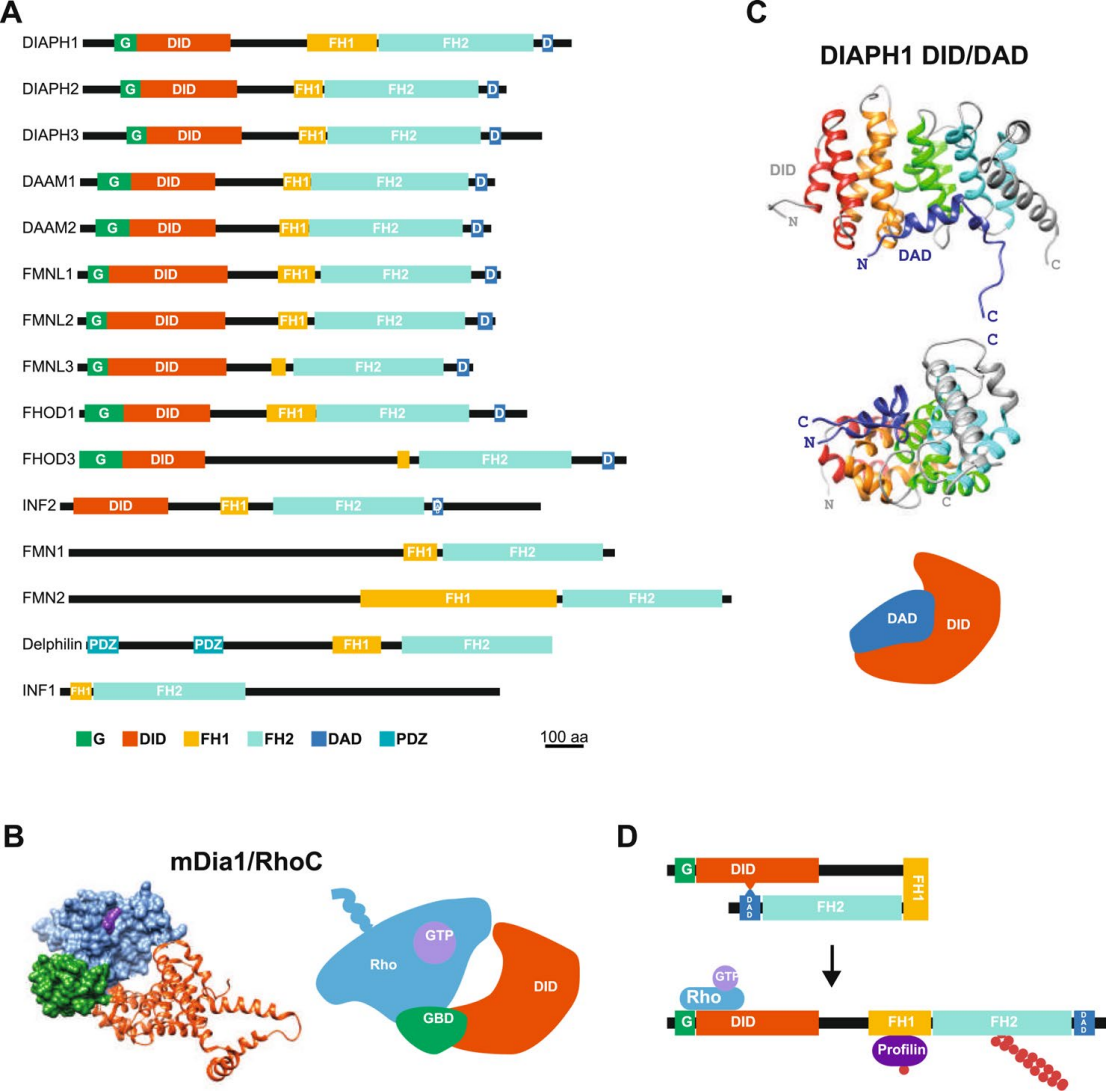
Structure of human formins and regulation of DIAPH activity by Rho family GTPases. **A** Domain organization of the fifteen human formins. **B** Structure of the complex of the DID of mDia1 with RhoC (PBD: 8FG1). The illustration shows the interaction of the RhoC with the GTPase-binding domain, which consists of the G region and the N-terminal half of the DID. **C** Structure of the DID-DAD complex of DIAPH1 (PBD: 8FG1). The illustration depicts the interaction of DID with DAD. **D** Regulation of DIAPH formins. The DID-DAD interaction maintains the formin in a closed, inactive conformation. Binding of a specific GTP-loaded Rho family GTPase to the N-terminal region of the formin opens the molecule, activating it. The FH1 domain recruits profilin, which supplies the FH2 domain with G-actin for actin filament elongation. DAD, diaphanous autoregulatory domain; DID, diaphanous inhibitory domain; G, GTPase-binding region; GBD, GTPase-binding domain; FH1, formin homology 1 domain; FH2, formin homology 2 domain; PDZ, PSD95/DLG/ZO-1

Of the fifteen formins in humans, eleven (DIAPH1-3, DAAM1-2, FMNL1-3, FHOD1, FHOD3 and INF2) contain a diaphanous inhibitory domain (DID) and a diaphanous autoregulatory domain (DAD), which are located N- and C-terminally to the core region, respectively (Fig. 2A). The DID consists of a tandem arrangement of a variable number of armadillo repeats (ARMs), each containing three α-helices [7]. In mDia1, the mouse ortholog of human DIAPH1, DID comprises four complete ARMs and a truncated fifth ARM [8, 9]. The DAD is a short sequence featuring an amphipathic α-helix followed by a stretch rich in basic residues [9, 10]. The DAD has a dual function: it regulates the activity of mDia1 and collaborates with FH1/profilin and the FH2 domain in actin nucleation [11].

DID- and DAD-containing formins, except for INF2, possess an extension of 100–130 amino acids N-terminal to the DID, known as the GTPase-binding (G) region (Fig. 2A). In mDia1, the small GTPase RhoC binds to the GTPase-binding domain (GBD), which consist of a bipartite surface formed by the G region and part of the DID. The switch regions of Ras-like GTPases alter their conformation in response to bound GTP or GDP nucleotides [12]. The switch I region of RhoC-GTP interacts exclusively with the G region, and the switch II region, which is invariant among the RhoA–C, Cdc42, and Rac 1–3 GTPases, contacts the G region and ARMs 1–2 of the DID (Fig. 2B) [8]. The mDia1 DAD interacts with ARMs 2–5 (Fig. 2C), typically maintaining the molecule in an autoinhibited conformation (Fig. 2D) [8, 10]. Due to the partial overlap between their binding sites, Rho binding displaces the DAD, releasing the formin from its closed, autoinhibited conformation to an open, active conformation with the FH1 and FH2 domains exposed (Fig. 2D) [8–10].

Sequence alignment analysis indicated that the G region of DIAPH, DAAM, FMNL and FMN is similar to that of mDia1 [13, 14]. However, FHOD members exhibit a ubiquitin superfold similar to that of the Ras GTPase-binding domain of the serine/threonine kinase cRaf-1 [15]. Although the function of the formins with a G segment is related to Rho-family GTPase activation, it is not clear whether all of these proteins are regulated directly by Rho family GTPase binding. For instance, in the case of DAAM, the interaction of the DAD with the PDZ domain of Disheveled releases DAAM from its autoinhibitory conformation, leading to RhoA activation [13].

## Structure and activity of the formin INF2

**Fig. 3:**
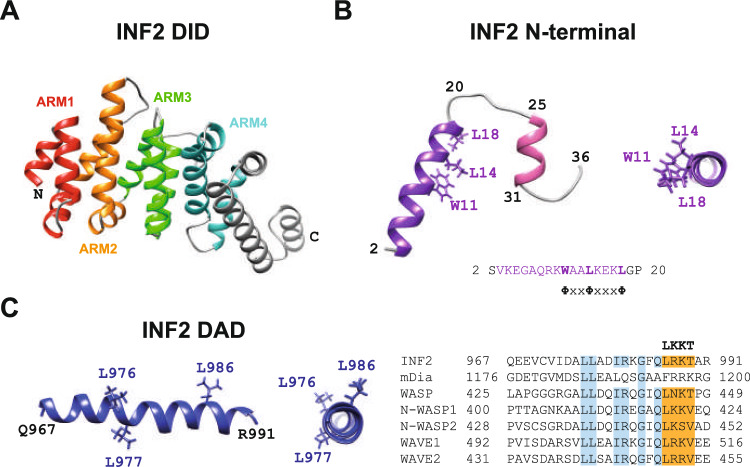
Structure of the regulatory domains of INF2. **A** Predicted structure of the INF2 DID according to AlphaFold. **B** Structure of the N-terminal extension of INF2 (amino acids 2–36) as determined by NMR of the isolated peptide (top, left panel; PBD: 9FJW). The orthogonal projection of the first helix, which contains the CaM-binding site, is also shown (top, right panel). Alignment of the amino acid sequence of the N-terminal extension of INF2 and a consensus CaM and centrin-binding site (bottom panel). The critical residues W11, L14 and L18 involved in CaM and centrin binding are indicated. φ indicates a hydrophobic amino acid, and X represents any amino acid. **C** Longitudinal and orthogonal views of the α-helical structure of the INF2 WH2/DAD sequence as determined by NMR of the isolated INF2 967–991 peptide (left panel, PBD: 9G7T). Alignment of the WH2/DAD sequence of INF2 (Q27J81) with the DAD of mDia1 (O8808) and the WH2 of the indicated panel of proteins: WASP (P42768), N-WASP (O00401), WAVE1 (Q92558) and WAVE2 (Q9Y6W5). The three conserved L residues in the WH2 motif are indicated in the structure (top panels). In the alignment, the actin-binding LKKT motif is shaded in orange, and all other amino acid identities are shaded in blue. ARM, armadillo repeat

INF2 is expressed as two isoforms, INF2-1 and INF2-2, which arise from alternative splicing and differ in their carboxyl termini. INF2-1 (also known as INF2-CAAX) has 1,249 amino acids and ends with a consensus CAAX box that is farnesylated, targeting it to the endoplasmic reticulum (ER). INF2-2 (also known as INF2-nonCAAX) has a C-terminal 9-amino acid sequence containing basic residues alternative to the 18-amino acid sequence of INF2-1 and is cytosolic [16]. The INF2 DID is predicted to be organized into four complete ARMs and one incomplete ARM (Fig. 3A), as is the organization in the mDia1 DID [8, 9]. Like other formins, the primary activity of INF2 is to catalyze the formation of F-actin through its FH2 domain, which also binds to and regulates microtubule stability.

The domain organization of INF2 includes several specific features:

1. The N-terminal extension of INF2 is shorter than that of other DID- and DAD-containing formins and consists of a 35-amino acid sequence. Instead of containing a G segment like other formins of this type, it is structured into two α-helices separated by a short loop (Fig. 3B) [17]. The lack of a G region suggests a mechanism of activation for INF2 distinct from that of mDia1 and other formins. However, similar to other formins in this group [18, 19], INF2 activity increases upon deletion of an N-terminal region containing the DID, suggesting that it can also fold in an autoinhibited conformation [20].
2. The INF2 DAD contains a Wiskott-Aldrich syndrome homology 2 (WH2) motif [21], a 17–20 amino acid sequence with high affinity for G-actin [22]. The INF2 DAD contains most of the residues conserved in canonical WH2 sequences, including a C-terminal LRKT sequence that fits the consensus LKKT motif of actin binding [22]. Conversely, the mDia1 DAD lacks an LKKT motif (Fig. 3C). Crystallographic studies revealed that, when bound to G-actin, WH2 motifs fold into an N-terminal α-helix that binds a hydrophobic cleft at the barbed end of G-actin and a C-terminal segment with an LKKT motif that extends along the G-actin surface [22]. In solution, INF2 WH2 adopts a long α-helical structure that includes the LKKT motif (Fig. 3C), suggesting that the C-terminal region of INF2 WH2 probably changes its structure upon binding to G-actin. The WH2 motif grants INF2 the ability to sever and depolymerize actin filaments in vitro, a function that could be exploited in cells to rapidly amplify filament number and create short and transient actin filaments [21, 23, 24].

## Regulation of INF2

**Fig. 4:**
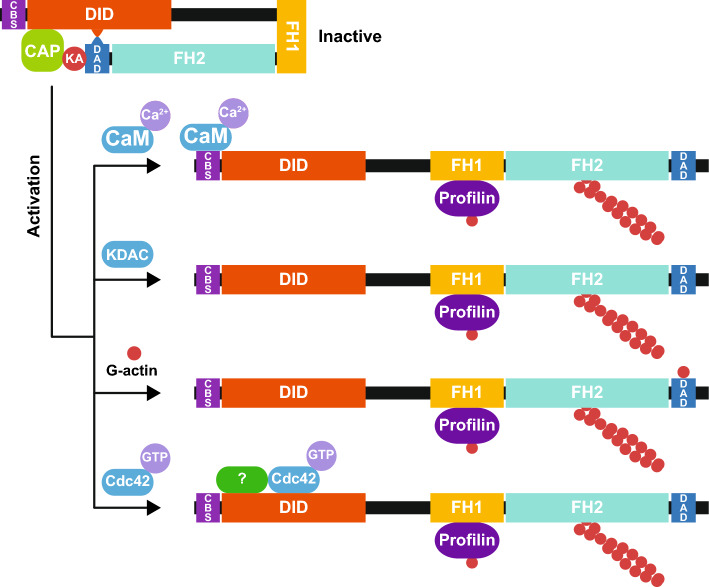
Proposed mechanisms of INF2 regulation. INF2 adopts an inactive conformation through interaction between DID and the DAD, facilitated by CAP/KAc-actin. INF2 activation occurs via the binding of Ca^2+/^CaM (or centrin) to the CaM and centrin-binding site of INF2, which is present in the N-terminal extension. Other proposed mechanisms of activation involve deacetylation of KAc-actin by a KDAC (potentially HDAC6), binding of G-actin to the DAD, and association of the Rho family Cdc42 GTPase with the DID. The latter association requires mediation by as yet unidentified protein(s). CaM, calmodulin; CAP, cyclase-associated protein; CBS, calmodulin and centrin-binding site; DAD, diaphanous autoregulatory domain; DID, diaphanous inhibitory domain; FH1, formin homology 1 domain; FH2, formin homology 2 domain; KA, lysine-acetylated actin; KDAC, lysine deacetylase

INF2 activity is negatively regulated by a complex of cyclase-associated protein (CAP)−1 or −2 [25] and lysine-acetylated actin (KAc-actin) [26, 27], and positively regulated by calmodulin (CaM) [17, 28]. Lysine deacetylases (KDACs) [29, 30] —such as HDAC6 [26, 27]—, G-actin [31], and Cdc42 [20, 32] are other potential INF2 regulators (Fig. 4).

### CAP/KAc-actin

In vitro, the DID-DAD interaction in INF2 is approximately 70-fold weaker (K_d_ = 16–28 μM) [26, 33] than that in mDia1 (K_d_ = 250–280 nM) [10, 34, 35]. Therefore, although purified DID and DAD-containing fragments of INF2 can interact directly in vitro, the affinity is insufficient for effective autoinhibition. Notably, the binding of KAc-actin enhances the strength of the INF2 DID-DAD interaction by approximately five-fold [27]. The WH2 motif of CAP binds to the INF2 N-terminal 420-amino acid segment containing the DID, while KAc-actin interacts with the INF2 WH2/DAD [26]. Thus, the CAP/KAc-actin complex appears to act as a bridge between DID and DAD, strengthening their interaction and contributing to maintaining INF2 in an inhibited conformation. Double KO of CAP1 and CAP2 in mice impairs dendritic spine maturation in hippocampal neurons [36]. Notably, while INF2 overactivation produced a similar effect in control mice, INF2 KD rescued the spine defects in double CAP KO mice [36]. The finding suggests that INF2 becomes deregulated in the absence of CAP, supporting CAP’s negative role on INF2 activity.

It is hypothesized that the acetylated lysine residues of KAc-actin are not part of the direct binding interface but that their acetylation results in conformational changes that enhance binding to the INF2 WH2 motif. Lysine (K)-to-glutamine (Q) mutation, which mimics acetylation at specific lysine residues, revealed that the K50Q and K61Q actin mutants confer inhibitory effects on actin polymerization by INF2 when coupled to CAP both in biochemical assays and in cells. However, the DID-DAD interaction mediated by CAP-K50Q in INF2 is still 20-fold weaker (K_d_ = 5.4 μM) than the direct DID-DAD interaction in mDia1 [26]. This discrepancy suggests that CAP-K50Q may not fully reflect the affinity of endogenous CAP/KAc-actin for INF2. Alternatively, while the CAP/KAc-actin complex does reinforce the DID-DAD interaction, additional regulatory mechanisms might be necessary to maintain INF2 in an autoinhibited state.

Lysine acetylation of proteins is catalyzed by lysine acetyltransferases (KATs), which transfer the acetyl group from acetyl-CoA to the ε-amino group of an internal lysine residue [29, 30]. There are more than 20 KAT family members in mammals, but the identity and means of regulation of the KAT responsible for acetylating actin to form the inhibitory CAP/KAc-actin complex remain unknown.

### Ca^2+^ levels: CaM and centrins

CaM is a Ca^2+^ sensor present in all eukaryotic cells that binds to a wide variety of proteins and regulates numerous cellular processes [37]. CaM has four canonical EF-hand motifs that mediate Ca^2+^ binding, with two located at the N terminus (N-lobe) and the other two at the C terminus (C-lobe). Centrins 1–3, a family of Ca^2+^ sensors, similarly possess N-terminal and the C-terminal lobes with EF-hand motifs [38]. CaM and centrins share the same binding site in INF2, which is in the first α-helix of the short N-terminal extension of INF2 [17]. Residues W11, L14 and L18 of INF2 are important for CaM and centrin binding, with W11 being the most critical [17, 39] (Fig. 3B).

In response to a transient increase in cytosolic Ca^2+^ levels, INF2 rapidly associates with Ca^2+^/CaM, leading to profound remodeling of the actin cytoskeleton [28, 40, 41]. Notably, inactivation of the CaM and centrin-binding site of INF2 completely impairs this response [17]. Although centrin binding to INF2 was not analyzed in these studies, it is likely that centrins also play a role in actin remodeling. Therefore, the relative contributions of CaM, centrins, and other EF hand-containing Ca^2+^ sensors to Ca^2+^-induced INF2 activation remain to be determined.

### Lysine deacetylase (KDAC)

Lysine deacetylation of proteins is catalyzed by approximately 20 KDACs, which include histone deacetylases (HDACs) and sirtuins [29, 30]. HDAC6 is a candidate for the KDAC responsible for deacetylating KAc-actin since its exogenous expression increases actin polymerization by INF2 [27]. However, as other deacetylases have not been assayed, it remains unclear whether this effect is specific to HDAC6. Treatments that increase cytosolic Ca^2+^ levels lead to KAc-actin deacetylation, resulting in decreased INF2 binding [27]. Therefore, the activation of the KDAC responsible for KAc-actin deaceylation could place INF2 in its active state by disrupting the CAP-KAc-actin inhibitory interaction.

### G-actin

The ability of purified full-length INF2 to accelerate actin polymerization was comparable to that of ΔDID INF2 in biochemical assays involving the addition of only INF2 and actin monomers. Moreover, the inclusion of proteins such as profilin or the WH2 domain of WASP in the assay decreases the activity of full-length INF2, but not that of ΔDID INF2 [31]. These in vitro experiments suggest that G-actin plays a role in disfavoring the autoinhibitory INF2 DID-DAD interaction. The C-terminal region of INF2, which contains the WH2/DAD, binds G-actin with an affinity (K_d_ = 78 nM) comparable to that of K50Q-actin (K_d_ = 63 nM**)** and 2.7-fold greater than that of CAP/K50Q-actin complex (K_d_ = 211 nM) [26]. Given their relative affinities for the WH2/DAD of INF2, unmodified G-actin could outcompete the in vitro binding of CAP/KAc-actin to the INF2 DAD, releasing INF2 into an active state. This makes INF2 well suited to act as a sensor of subtle physiological fluctuations in free cytosolic G-actin levels, whereby an increase or decrease in G-actin levels modulates INF2 activity and thereby controls actin homeostasis [42]. The high concentration of G-actin binding proteins, such as profilin and thymosins, in the cytosol could significantly reduce free G-actin levels, thereby modulating INF2 activity. Increased levels of G-actin have been proposed as a potential mechanism for INF2 activation in ischemia-induced dendritic actin reorganization [43].

### The Rho-family Cdc42 GTPase

Cdc42 potentiates INF2-mediated functions in membrane trafficking in T cells and epithelial cells. Conversely, Cdc42 depletion or expression of dominant negative Cdc42 has the opposite effect [20, 32]. Despite lacking a G region, an INF2 fragment containing DID and its N-terminal extension associated with Cdc42 in cell extracts as demonstrated by GST pull-down assays [20, 32]. However, no binding was observed when pull-down experiments were performed using purified Cdc42 [31], suggesting that the interaction is not direct but rather mediated by an additional protein present in cell extracts. Nevertheless, this result does not rule out a low-affinity interaction between the INF2 DID and Cdc42 in the absence of such a protein factor.

The switch II region, which is identical in RhoA-C, Rac and Cdc42 [12], and the high degree of identity and conservation between the ARMs 1–2 of INF2 and those of mDia1 [44], which interact with the switch region II of RhoC [8], make it plausible that the switch II region of Cdc42 interacts with ARM 2 of INF2. A possible mechanism is that a Cdc42-binding protein binds INF2, bringing Cdc42 into proximity to the DID, allowing Cdc42 to interact. This mechanism is reminiscent of the two-step mechanism proposed for Rho binding to mDia1, where Rho first binds to the G domain with high affinity and then to DID [10]. CaM, which binds to the N-terminal extension of INF2 and is known to interact with the switch I region of Cdc42 while leaving switch II free [45], is a good candidate for facilitating this interaction. Another candidate could be IQGAP1 [46], which binds Cdc42 and interacts with the C-terminal region of INF2 [47]. In summary, although a functional and biochemical link exists between INF2 and Cdc42, the exact mechanism of this interaction remains unclear.

## Signaling pathways regulated by INF2

### Ca^2+^ signaling

**Fig. 5:**
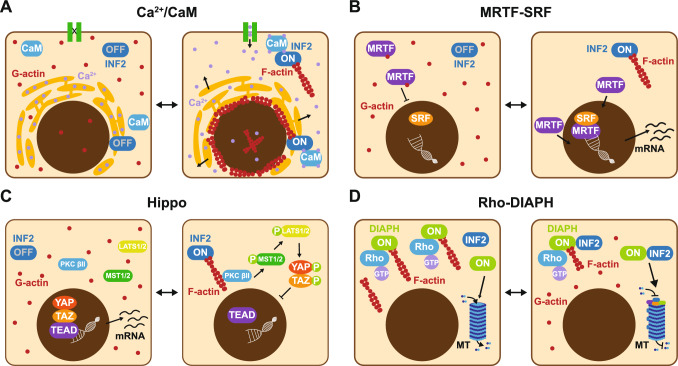
Signaling pathways in which INF2 has been implicated. **A** Ca^2+/^CaM signaling. Increased cytosolic Ca^2+^ levels, whether from external entry or intracellular release, activate INF2 through the binding of Ca^2+^/CaM to the N-terminal extension. This activation leads to massive actin polymerization, resulting in the formation of a characteristic F-actin ring around the nuclear envelope. Elevated Ca^2+^ levels in the nucleus also activate INF2, resulting in the transient formation of thin actin filaments. **B** MRTF-SRF signaling. INF2-mediated actin polymerization depletes G-actin from the cytosol, allowing MRTF to enter the nucleus and associate with the transcription factor SRF. The MRTF/SRF complex directs the transcription of target genes. **C** Hippo pathway. Activated INF2 polymerizes specialized actin filaments, facilitating the recruitment of PKC βII, which phosphorylates the transcription factors YAP and TAZ. This phosphorylation prevents the nuclear translocation of these proteins and their association with TEAD, thereby inhibiting the transcriptional activation of target genes. **D** Rho-DIAPH signaling. The DID of INF2 interacts with the DAD of DIAPH formins, inhibiting actin polymerization via DIAPH. Additionally, INF2 and DIAPH collaborate in stabilizing microtubules in response to Rho signaling

Mechanical forces (such as direct force application and shear flow), physiological ligands for G protein-coupled receptors (e.g., thrombin and lysophosphatidic acid) and drugs (e.g., A23187 and ionomycin) increase intracellular Ca^2+^ levels, triggering rapid and transient remodeling of the actin cytoskeleton [28, 41, 48, 49]. This fundamental response in mammalian cells leads to an increase in F-actin within the cytoplasm, perinuclear region and nucleus, and it requires INF2 expression (Fig. 5A). Cytoplasmic actin remodeling causes transient organelle immobilization, facilitates the reorganization of actin during cell cortex repair, cell spreading and wound healing, and induces long-lasting changes in gene expression. The perinuclear F-actin ring protects the nucleus from mechanical damage [50], while nuclear F-actin is involved in regulating chromatin organization and dynamics [49]. The inner nuclear transmembrane protein SUN associates with INF2, a connection that is essential for nuclear actin assembly and the clustering of active RNA polymerase II in response to Ca^2+^ elevation [51]. Another example of INF2 activation by Ca^2+^ is seen in melanoma cells, where the cation-selective channel Piezo1 elevates Ca^2+^ levels in response to cell stretch. This increase in Ca^2+^ triggers actin remodeling and de-adhesion, driving the mesenchymal-to-amoeboid transition in an INF2 expression-dependent manner [52].

### Actin-MRTF-SRF transcriptional circuit

The serum response factor (SRF) is a widely expressed mammalian transcription factor regulated by myocardin-related transcription factor (MRTF) coactivators. MRTF forms a reversible complex with G-actin, remaining in an inactive state in the cytosol. When the cytosolic G-actin concentration decreases due to massive actin polymerization, MRTF enters the nucleus and associates with SRF to direct the transcription of numerous genes involved in cytoskeleton regulation, transcription, and cell growth and metabolism [53]. In response to fluctuations in the G-actin level, INF2 modulates MRTF/SRF complex formation in the nucleus and, subsequently, its transcriptional activity (Fig. 5B) [28, 54]. This regulation is exemplified by the expression of the *α-tubulin acetyltransferase (α-TAT1*) gene, which is responsible for α-tubulin acetylation: INF2 KO cells exhibit negligible *α-TAT1* mRNA expression due to cytosolic G-actin accumulation, while cells expressing constitutively active INF2 overexpress *α-TAT1* [54].

### Hippo pathway

Activation of the Hippo pathway promotes cytoplasmic retention and proteasomal degradation of the transcriptional coregulators YAP and TAZ, preventing their association with the transcription factors TEAD 1–4 to regulate growth, proliferation, and cell fate decisions. Upstream signals regulating the Hippo pathway include cell polarity, mechanical cues (e.g.: extracellular matrix stiffness, cell geometry, liquid flow, and tension), cell density, soluble factors (e.g.: GPCR ligands such as thrombin, LPA, and sphingosine-1-phosphate) and stress signals (e.g.: hypoxia, ER stress, energy stress, osmotic stress, and heat stress). Some of these stimuli converge on the actin cytoskeleton as a key upstream mediator of the Hippo pathway [55]. Therefore, unlike the MRTF-SRF pathway, the Hippo pathway is not simply regulated by G-actin levels; it also depends on specialized F-actin structures [56]. Mechanical force and stimulation of specific GPCRs are stimuli that activate the Hippo pathway, triggering a transient increase in cytosolic Ca^2+^ levels. INF2 is crucial for Hippo pathway regulation in a human glioblastoma cell line [57, 58]. Although the exact role of F-actin in this process is unclear, it has been proposed that Ca^2+^/CaM-stimulated INF2 polymerizes specialized F-actin structures in the cell cortex, providing a scaffold to recruit PKC βII and other machinery involved in Hippo signaling activation (Fig. 5C) [57, 58].

### Rho GTPase-DIAPH signaling

The DAD of DIAPH interacts with the DID of INF2, antagonizing DIAPH-mediated actin polymerization in cells and in vitro with purified components (Fig. 5D) [33]. This interaction was initially puzzling since, in principle, both formins should be in their open active conformation in order to interact. Further research revealed that INF2 is proteolytically cleaved by cathepsin proteases in two halves: an N-terminal fragment (amino acids 2–547) containing the N-terminal extension, DID, the FH1 domain and part of the FH2 domain, and a C-terminal fragment containing the rest of the molecule [59]. The N-terminal INF2 fragment may mediate interaction with the DIAPH DAD, although how this reduces DIAPH-mediated actin polymerization remains unclear.

Additionally, the Rho-DIAPH axis controls microtubule dynamics [60]. The absence of INF2 blocks the promotion of microtubule stabilization by Rho-activated DIAPH. INF2 fragments containing the FH2 domain bind and stabilize microtubules against depolymerization in vitro and promote microtubule stabilization in cells, even without DIAPH [47]. This finding suggested that INF2 acts downstream of DIAPH, and that microtubule stabilization might be regulated by the DIAPH DAD-INF2 DID interaction (Fig. 5D).

INF2’s regulation of multiple signaling pathways suggests that it may serve as a coordinator. reprogramming cellular behavior according to specific contexts and demands. For instance, the simultaneous increase in MRTF activity and decrease in YAP-TAZ signaling could promote cell adhesion while inhibiting migration, thereby balancing motility and stability. This coordination may be critical for maintaining cellular integrity, and disruptions in these pathways could contribute to disease processes.

## Cellular processes involving INF2

### Membrane traffic

**Fig. 6:**
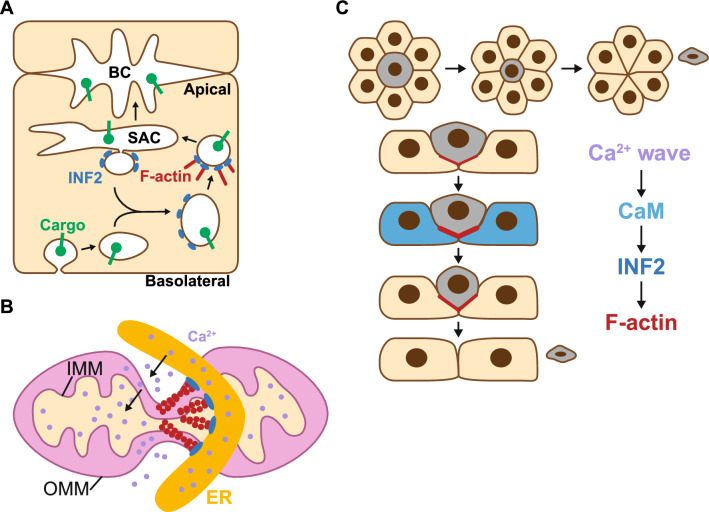
Cellular functions of INF2. **A** INF2 facilitates the transcytotic transport of basolateral cargo proteins to the apical membrane of hepatic cells. BC, bile canaliculus; SAC, subapical compartment. **B** INF2 promotes mitochondrial fission by inducing constriction of the outer (OMM) and inner (IMM) mitochondrial membranes. ER, endoplasmic reticulum. **C** In response to a wave of increased Ca^2+^ levels triggered by a nearby apoptotic or cancerous cell (in gray), this cell is extruded by surrounding cells in an INF2 expression-dependent manner. The schematic illustrates XY (top) and XZ (bottom) views of the cell monolayer during the extrusion process. CaM, calmodulin

INF2 plays a crucial role in specialized pathways of intracellular vesicular transport (Fig. 6A). INF2 is associated with transport vesicles as they move to the plasma membrane, decorating the growing end of short actin filaments that appear to propel vesicle movement in a Cdc42-controlled process [20, 32]. Constitutive RhoA activation in mouse podocytes leads to proteinuric kidney disease and FSGS, with foot process effacement, prominent intracellular stress fibers, and altered distribution of the slit diaphragm proteins nephrin and podocin [61, 62]. Expression of INF2, which associates with podocin and nephrin, counteracts these effects probably by modulating RhoA-activated mDia1 activity, preserving protein trafficking [63]. Supporting the role of INF2 in vesicular trafficking, INF2 silencing in zebrafish results in developmental anomalies and nephrin mislocalization [64]. This role probably involves INF2’s interaction with dynein light chain 1 [65], a component of the transport machinery. Thus, INF2 regulates specialized vesicular trafficking, in addition to polarized epithelial cells and T cells, in podocytes.

### Mitochondrial dynamics

Mitochondria undergo regulated fusion and fission to maintain a dynamic network [66, 67]. Mitochondrial division involves the fission of both the inner and outer mitochondrial membranes (IMM and OMM). The dynamin-like GTPase Drp1 translocates from the cytosol to the OMM, providing the force for OMM fission [68]. ER-bound INF2 promotes mitochondrial fission by inducing initial constriction before Drp1 is concentrated in the mitochondria [69]. In collaboration with the actin-nucleating protein Spire1C [70], INF2 promotes actin polymer assembly at ER-mitochondrial contacts and, together with myosin II, stimulates Drp1 recruitment and fission [71–73]. Additionally, INF2-induced actin polymerization increases mitochondrial matrix Ca^2+^ levels, activating IMM constriction (Fig. 6B) [41]. Mitochondrial fusion is also impaired in INF2 KO cells [74], though it is yet to be determined whether this effect is direct or merely a consequence of the reduced fission rates.

### Cell extrusion

The epithelium removes cells as part of normal cell homeostasis or to eliminate aberrant cells without compromising barrier function, through a process known as cell extrusion. This process involves successive remodeling steps that lead to cell expulsion while maintaining the epithelial barrier in a sealed state [75]. Cell extrusion is coordinated by Ca^2+^ waves originating from the extruding cell that rapidly propagate to neighboring cells. INF2 is essential for the formation of a supracellular actomyosin ring around the extruding cell, which pushes the cell out apically and brings neighboring cells together to maintain the sealed state of the epithelial barrier (Fig. 6C) [76]. It remains to be determined whether this function depends on the integrity of the CaM and centrin-binding site in the N-terminal extension of INF2 and the specific Ca^2+^ sensor involved.

## INF2-linked disorders

### Focal segmental glomerulosclerosis

Focal segmental glomerulosclerosis (FSGS) is a kidney lesion characterized by sclerosis in part of some glomeruli [77]. The common denominator in the diverse pathogenic processes leading to FSGS is podocyte injury, which compromises the selectivity of the glomerular filtration barrier, resulting in proteinuria. The condition often progresses to nephrotic syndrome and chronic kidney disease and, in severe cases, culminates in end-stage kidney disease. Whereas autosomal recessive FSGS typically manifests within the first year of life and tends to be fully penetrant, with rare asymptomatic cases, autosomal dominant FSGS is more prevalent in older children, adolescents, and adults and displays incomplete penetrance, with some family members harboring the pathogenic variant without showing disease signs [78]. Variants in the *INF2* gene cause autosomal dominant FSGS (FGS5, OMIM: 613237), making INF2 the major cause of autosomal dominant FSGS, accounting for up to 17% of cases [79, 80].

### Charcot–Marie–Tooth disease

Charcot–Marie–Tooth disease (CMTD) is an inherited neurological disorder affecting peripheral nerves, leading to progressive distal muscle weakness. Most types of CMTD are inherited in an autosomal dominant manner, but there are also autosomal recessive and X-linked forms. Symptoms usually appear in the first or second decade, with severity ranging from severe deficits in early childhood to milder symptoms in late life [81]. Over 100 genes are associated with various forms of CMTD [82]. After identifying *INF2* gene mutations as a cause of FSGS, *INF2* mutations were found in approximately 75% of patients with combined FSGS and CMTD (CMTDIE, OMIM: 614455) [83]. In these patients, CMTD symptoms emerge during childhood, and renal symptoms appear earlier than in patients with FSGS alone. Pathogenic INF2 affects Schwann cell morphology and leads to axonal involvement; however, it remains unclear whether the axonal effects result directly from neuronal impact or are secondary to changes in Schwann cell architecture [84].

## Pathogenic human INF2 variants and in silico analysis of their pathogenicity

**Fig. 7:**
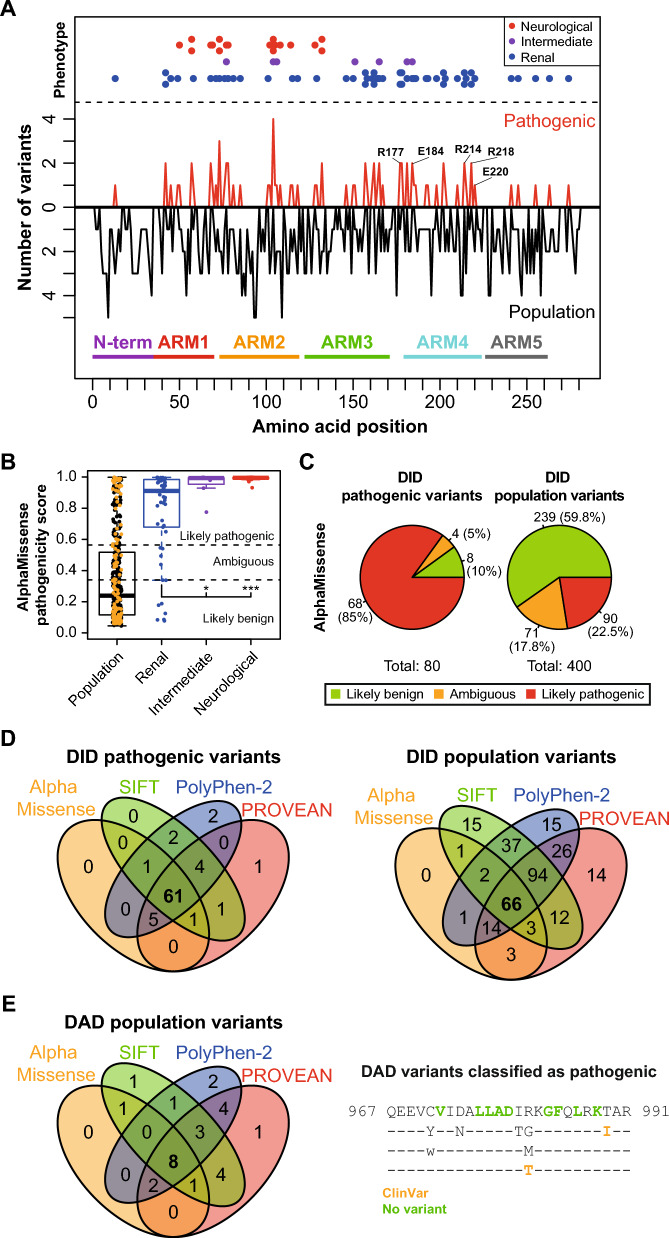
Distribution and analysis of human INF2 DID variants. **A** The number of human INF2 variants reported as pathogenic (red) and those annotated in public databases but not yet studied for disease implications (black) are shown relative to their position in the INF2 sequence. The predominant phenotypes (renal alone, renal plus neurological/neurological alone, or intermediate) are indicated (top panel). “Intermediate” refers to mutations that result in only FSGS in some patients but in FSGS combined with CMTD in others. The N-terminal extension and the armadillo repeat (ARM) arrangement of the DID are also illustrated (bottom panel). **B** Boxplots displaying the pathogenicity scores assigned by AlphaMissense to INF2 DID mutations associated with renal-only, renal plus neurological or neurological-only, and intermediate phenotypes, as well as those variants annotated in public databases with not known disease implications. Variants found in ClinVar are highlighted in orange. *, p < 0.05; *** p < 0.001; Mann–Whitney test. Variants are categorized as likely pathogenic, likely benign, or ambiguous based on AlphaMissense thresholds. **C** AlphaMissense pathogenicity prediction for 80 INF2 DID variants reported to be pathogenic (left panel) and 400 DID variants annotated in public databases without no disease involvement (right panel). **D** Venn diagram illustrating the overlap in pathogenicity predictions for 80 human INF2 variants reported as pathogenic and 400 variants annotated in public databases, based on the AlphaMissense, SIFT, PolyPhen-2, and PROVEAN algorithms. **E** Venn diagram showing pathogenicity predictions from the AlphaMissense, SIFT, PolyPhen-2, and PROVEAN algorithms for the 32 human INF2 WH2/DAD variants annotated in public databases (left panel). The eight mutations classified as likely pathogenic by all four predictors are indicated (right panel). The two variants from this group annotated in ClinVar are shown in orange. INF2 DAD residues with no substitutions are highlighted in green in the wt INF2 sequence. Only mutations predicted as “likely pathogenic” by AlphaMissense, “deleterious” by PROVEAN, “damaging” by SIFT, or “probably damaging” by PolyPhen-2 were considered for both panels (**D**, **E**). ARM, armadillo repeat

At least 80 mutations have been reported to be pathogenic in INF2, with most supported by familial studies [85]. These mutations map to the segment spanning amino acids 2–281, which includes the DID (Fig. 7A). Depending on the specific mutation, FSGS manifests with or without CMTD, with only a few reported cases in which INF2 mutation exclusively causes CMTD. In intermediate cases, specific mutations result in only FSGS in some patients but in FSGS combined with CMTD in others [85]. The pathogenic mutations of INF2 are distributed throughout the DID (Fig. 7A, Table S1), with leucine substituted by proline being the most frequent mutation (Table S2). Mutations causing CMTD tend to be concentrated in ARMs 1–2, while those causing FSGS are distributed throughout all the ARMs. Previous in silico analyses using Rosetta software (Cyrus Bench®, https://cad.cyrus bio.com) —which predicts the impact of single amino acid substitutions on protein stability and structure by calculating the variation of free energy between the folded mutant and the wild-type protein— indicated that pathogenic mutations destabilize the architecture of INF2 DID. Moreover, mutations associated with both FSGS and CMTD were found more destabilizing than those causing isolated FSGS [85, 86].

AlphaMissense (github.com/google-deepmind/alphamissense) is an artificial-intelligence tool designed to predict the pathogenicity of missense variants generated from single-nucleotide replacements in protein-coding DNA regions. AlphaMissense assigns a score between 0 and 1, indicating the probability of a variant being likely benign, ambiguous or likely pathogenic. For INF2, AlphaMissense predicts 7329 possible missense mutations, of which 1647 are located within the region encoding amino acids 2–281 (which contains the DID) (Table S1), and 5682 are located elsewhere (amino acids 282–1249) in the INF2 molecule (Table S3). A higher proportion of mutations in DID (576/1,647; ~ 35%) are classified as likely pathogenic (Table S1) compared to the rest of the molecule (897/5682; ~ 16%) (Table S3). This suggests that the DID region is twice as prone to deleterious missense mutations as the rest of INF2 (Fig. S1A). Since there are no reports of familial studies showing that the mutations outside the DID cause disease, the variants predicted to be pathogenic may either not be genuinely pathogenic or may require a second hit in another gene for the disease to manifest.

Analyzing the group of 80 reported pathogenic DID variants (“the pathogenic group”) with AlphaMissense reveals notable trends. Variants associated with neurological disease have a higher median pathogenicity score than those causing only FSGS, with intermediate scores for variants with a mixed phenotype (Fig. 7B). AlphaMissense classifies 85% of the variants as likely pathogenic (Fig. 7C, left panel). This percentage is consistent with the values of 91.2%, 87.5% and 93.8% (Fig. S1B) which were respectively obtained from PROVEAN (provean.jcvi.org), which evaluates clusters of sequences homologous to the protein of interest; SIFT (sift.bii.a-star.edu.sg), which assesses sequence homology and the physicochemical similarity between alternative amino acids; and PolyPhen-2 (pfam.xfam.org), which relies on sequence homology and 3D structural information. Integrating the results from all four algorithms reduces the number of likely pathogenic mutations to 61 (Fig. 7D, left panel) and reveals that two variants within the pathogenic group, T215S and S263A, are likely benign (Fig. S1C and Table S1). These variants may be classified as variants of unknown significance, or they could contribute to disease only when combined with a second hit in another gene. The latter possibility is supported by the identification of variants of unknown significance in ROBO2 and LAMB2 —two genes associated with renal problems— in patients carrying the INF2 T215S and S263A mutations, respectively [87, 88]. Further research is needed to characterize these cases.

## Additional INF2 DID variants and analysis of their pathogenic potential

In addition to the reported pathogenic mutations in the INF2 DID, numerous variants in this domain are cataloged in public databases such as dbSNP (ncbi.nlm.nih.gov/snp), gnomAD (gnomad.broadinstitute.org), LOVD3 (lovd.nl), TOPMED (topmed.nhlbi.nih.gov) and ClinVar (ncbi.nlm.nih.gov/clinvar), the latter of which uniquely includes data on disease associations and clinical implications. Despite this, the study of these mutations in relation to disease remains largely unexplored, even though identifying which variants are truly pathogenic would greatly aid in diagnosis. The 400 mutations within this group (“the population group”) are distributed throughout the DID (Fig. 7A) and generally exhibit a low median pathogenicity score, suggesting that most are likely benign (Fig. 7B). Notably, AlphaMissense classifies 90 of these mutations as likely pathogenic (Fig. 7C, right panel), with concurrence from SIFT, PolyPhen-2 and PROVEAN analyses in 66 mutations (Fig. 7D, right panel; Fig. S1B, right panels; Table S1). Therefore, a subset of the mutations in the population group is likely to cause disease, such as the 26 out of these 66 variants that are annotated in ClinVar as being associated with pathological conditions (Table S4), or potentially contributes to mild disorders that have gone unnoticed. In summary, the 80 reported INF2 DID mutations probably underestimate the true number of disease-causing mutations in this region. Comprehensive genetic analysis of the association between these potential pathogenic variants and disease would facilitate more accurate diagnoses in novel cases of INF2-linked conditions.

## Potential pathogenic INF2 variants in the DAD

In addition to those in the INF2 DID, numerous mutations are cataloged in public databases, with only a handful documented in patients with kidney disease [89]. However, it remains uncertain whether these mutations cause the disease or are merely variants of unknown significance. Among the variants outside the DID, those mapping to WH2/DAD are particularly intriguing candidates for disease association due to the regulatory function of this region. There are 148 potential missense mutations in the DAD that could result from single nucleotide substitution. Of these, 32 variants are listed in public databases (Table S5). Notably, none of these affect the three L residues that are crucial for WH2 motifs or the basic amino acids of the LKKT motif, except for the R987N substitution, which is also observed in the WASP WH2 motif (Fig. 3C), and the R987W and R987Q mutations. Of the 32 mutations, 8 meet the criteria of the four algorithms to be classified as likely pathogenic, and 12 are annotated in ClinVar as possible cause of FSGS, CMTD or other genetic disorders. Two variants (R981T and T989I) complied with the four algorithms and are documented in ClinVar as occurring in individuals with disease (Fig. 7E, Table S5). Thus, it is plausible that, alongside mutations in the DID, specific variants within the DAD may also contribute to pathological conditions. This consideration could be significant for the genetic diagnosis of INF2-linked disease.

## Impact of pathogenic mutations on INF2 interactions

**Fig. 8:**
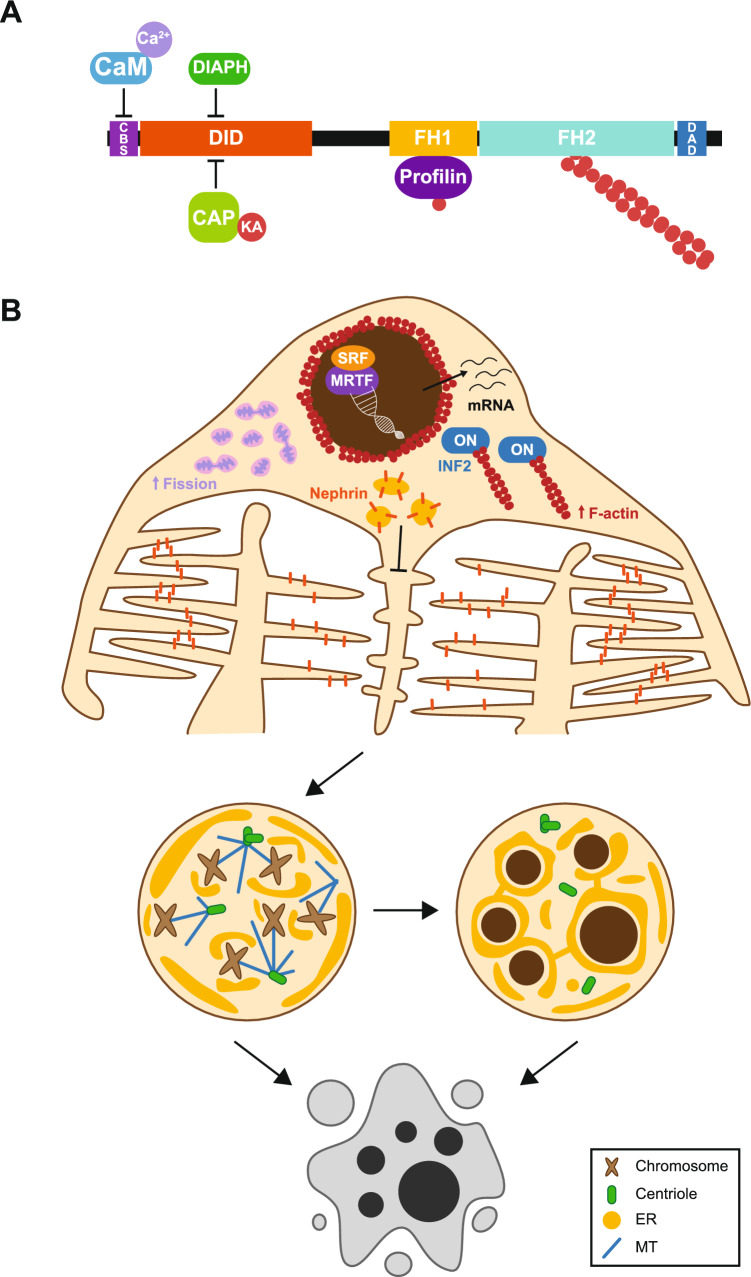
Effect of the pathogenic mutations on the regulatory interactions of INF2. **A** Pathogenic mutations in INF2 disrupt the binding of Ca^2+^/CaM to the calmodulin and centrin-binding site, the CAP/KAc-actin complex, and the DAD of DIAPH to the DID. CaM, calmodulin; CAP, cyclase-associated protein; CBS, calmodulin and centrin-binding site; DAD, diaphanous autoregulatory domain; DID, diaphanous inhibitory domain; FH1, formin homology 1 domain; FH2, formin homology 2 domain; KA, lysine-acetylated actin. **B** Model of podocyte loss during glomerular degeneration due to pathogenic INF2. Over time, the expression of pathogenic INF2 in podocytes results in progressive dysregulation of critical processes. These include impaired polarized transport of slit diaphragm proteins, dysregulated mitochondrial fission, and altered MRTF/SRF complex-mediated transcription. As a consequence, podocytes depolarize and attempt division, encountering severe mitotic abnormalities that lead to mitotic catastrophe and death, either immediately or after generating cells with nuclear aberrations

Pathogenic mutations disrupt the binding between the DID and the DAD of INF2 [90]. These mutations also interfere with the association with CAP/KAc-actin, probably by disrupting the interaction between CAP and the INF2 DID [27]. Consequently, INF2 is unable to adopt its autoinhibitory conformation and remains constitutively active (Fig. 8A). Expression experiments in cultured cells confirmed that pathogenic INF2 variants exhibit deregulated F-actin polymerization activity [27, 39, 48, 91].

Disease-linked mutations in the INF2 DID also impair the interaction with the DIAPH DAD, which is important for regulating DIAPH activity and maintaining proper actin dynamics (Fig. 8A) [33]. Although the binding site for CaM is outside the DID, pathogenic INF2 also shows impaired association with CaM (Fig. 8A) [48]. This suggests that these mutations may alter the DID structure, potentially obstructing the CaM-binding site. Alternatively, this could indicate that the DID of wt INF2, unlike that of pathogenic variants, facilitates efficient interaction with CaM. However, the latter possibility appears unlikely, as CaM binds robustly to the INF2 1–19 fragment, which contains only the CaM-binding site [17]. Consistent with the constitutive activation of pathogenic INF2, its activity mirrors the maximal activity of Ca^2+^/CaM-activated wild type INF2, suggesting that it is not influenced by decreased Ca^2+^/CaM binding [27, 48]. Nonetheless, given the extensive CaM interactome, which includes a wide variety of scaffold and regulatory proteins [92], the reduced binding of CaM might affect the recruitment of relevant proteins to the INF2 environment.

## Impact of pathogenic INF2 on cellular functions

### Aberrant membrane trafficking

Pathogenic INF2, unlike its wild type counterpart, fails to counteract the defects caused by the activation of the Rho-DIAPH axis in the trafficking of podocin and nephrin in human immortalized podocytes [63]. These mutations impair the interaction between INF2 and dynein light chain 1, and consistent with the importance of this interaction, endocytosed nephrin is misrouted from recycling endosomes to the degradation pathway in podocytes derived from R218Q knock-in (KI) mice [65] (Fig. 8B). Further confirming the effect of pathogenic INF2 on vesicular transport, altered nephrin distribution was observed in kidney organoids derived from induced pluripotent stem cells from a patient with the INF2 S186P pathogenic mutation [91]. Additionally, defects in lysosomal trafficking have been observed in HeLa cells expressing pathogenic INF2 [93].

### Abnormal mitochondrial division and dynamics

The expression of the constitutively active INF2 A149D mutant increased the frequency of mitochondrial constriction, resulting in increased fission and a significant reduction in mitochondrial length (Fig. 8B) [69, 94]. Pathogenic INF2 mirrors the effect of INF2 A149D on mitochondrial fission and length, and diminishes mitochondrial mobility, impairing the ability of mitochondria to interact and fuse, and reduces mitochondrial function [69, 93, 95]. Imbalances in mitochondrial division and fusion dynamics have been implicated in various pathological conditions, including aging, degenerative diseases, metabolic disorders and cancer [96, 97].

### Constitutive MRTF/SRF complex-mediated transcription

Pathogenic INF2 leads to deregulated actin polymerization [39, 48, 91], disrupting actin homeostasis and causing the accumulation of MRTF in the nucleus [39]. This results in sustained activation of MRTF/SRF complex-mediated transcription. The aberrant activation of this pathway extensively reprograms the transcriptome, altering the expression of numerous genes (Fig. 8B). This change includes genes involved in critical cellular processes, such as the actin cytoskeleton, cell adhesion, cell migration, response to stress, signaling, regulation of gene expression, and intracellular transport [39].

## Consequences of pathogenic INF2 expression on cell viability

Mitochondrial fission and fusion are critical events in mitochondrial and cellular health. Fusion allows healthy mitochondria to supply fresh components to compensate for dysfunctional ones while also facilitating mitochondrial autophagy, since elongated mitochondria are less susceptible to degradation. An imbalance in mitochondrial dynamics leads to sustained damage, causing dysfunction in cellular energy metabolism and contributing to disease development [98]. The transient activation of the MRTF/SRF transcriptional complex in response to specific stimuli results in dramatic remodeling of gene expression, helping the cell to adapt to new conditions. However, its sustained activation, as observed in cells expressing pathogenic INF2, can be detrimental to the cell [39]. Although the effects of pathogenic INF2 on other signaling pathways regulated by INF2, such as the Hippo pathway and the Rho GTPase-DIAPH signaling pathway, have not yet been explored, it is conceivable that these pathways may also be disrupted, potentially contributing to the pathogenesis of FSGS. Consequently, pathogenic INF2, through its constitutive actin polymerization activity, profoundly affects the cell by altering its cytoskeleton, mitochondrial homeostasis, organelle dynamics, and gene expression [39, 91, 93, 94] (Fig. 8B).

In nontransformed renal epithelial cells, pathogenic INF2 causes centrosome fragmentation, leading to the generation of supernumerary microtubule-organizing centers (MTOCs). These extra MTOCs form multipolar spindles, hindering the correct alignment of chromosomes at metaphase and resulting in delayed or completely blocked mitosis. When mitosis is delayed, the ER invades the spindle space and envelops the scattered chromosomes, culminating in the formation of nuclei with various aberrations, predominantly multiple micronuclei. In both scenarios —delayed or blocked mitosis— the cells ultimately die through an antiproliferative mechanism, known as mitotic catastrophe, and detach from the substrate [39]. The involvement of mitotic catastrophe in INF2-linked disease aligns with the hypothesis that it is a general mechanism underlying the progressive loss of podocytes in FSGS [99, 100], which is supported by the presence of multinucleated cells in the urine and glomeruli of patients [101–104].

The p53 tumor suppressor safeguards genomic stability in nontransformed cells. Activation of p53 results in its translocation into the nucleus, where it orchestrates transcriptional programs involved in DNA repair, cell cycle arrest, senescence and apoptosis [105]. In renal epithelial MDCK cells expressing pathogenic INF2, p53 activates caspase-3, a key effector of apoptosis, and is concentrated in the nucleus, where it alters the transcriptome. These observations suggest that mitotic catastrophe in these cells triggers cell death via p53-mediated apoptosis. In contrast, transformed cell lines such as HEK293T cells or HeLa cells are resistant to pathogenic INF2. The inhibition of p53 in HEK293T cells and its degradation in HeLa cells imply that the absence of active p53 protects them from death [39]. Therefore, the susceptibility of different cell types to pathogenic INF2 probably depends on the balance between p53-independent protective mechanisms and p53-mediated apoptosis.

## Animal models of INF2-linked FSGS

Neither INF2 KO nor INF2 R218Q KI mice exhibit renal pathology, indicating that normal INF2 is not essential for kidney development [91, 106]. When INF2 R218Q KI mice were subjected to acute kidney injury by perfusion with protamine sulfate, they did not develop overt FSGS. This treatment, however, impaired podocyte plasticity, preventing the restoration of normal podocyte architecture after perfusion with heparin sulfate, which neutralizes protamine [106]. This outcome suggests that repeated kidney injuries over time may be necessary to develop the FSGS phenotype in these mice. However, in response to puromycin aminonucleoside stress, INF2 R218Q-KI mice, but not INF2 KO mice, began to develop proteinuria after 3 days and showed signs of FSGS 8 weeks later [91]. This result indicates that INF2 R218Q KI mice stressed with puromycin aminonucleoside could serve as an appropriate animal model for studying INF2-linked FSGS.

## Potential strategies for combating INF2-linked disease

The primary effect of pathogenic INF2 mutations on INF2 activity is the deregulation of actin polymerization [39, 48, 91], which affects mitochondrial dynamics and MRTF/SRF complex-mediated transcription. This disruption leads to mitotic catastrophe-mediated cell death and detachment. In cultured cells, suboptimal doses of Latrucunlin B (LatB) correct MRTF/SRF complex-mediated transcription and dysregulation of mitochondrial dynamics. LatB binds to G-actin, blocking massive actin polymerization by pathogenic INF2 and inhibiting its adverse effects on mitochondrial dynamics. Additionally, LatB stabilizes the G-actin/MRTF complex in the cytosol, preventing MRTF from translocating into the nucleus. This dual action of LatB reduced nuclear abnormalities and cell death caused by pathogenic INF2 [39].

Pathogenic mutations in INF2 also disrupt the interaction of DID with dynein light chain 1, impairing dynein-mediated vesicular trafficking to the slit diaphragm and affecting the functionality of the filtration barrier [65]. As a potential therapeutic strategy for INF2-linked FSGS, pharmacological inhibitors that target actin polymerization —particularly that mediated by INF2— could be explored to restore actin homeostasis. Other strategies might include modulating MRTF/SRF complex activity or correcting dynein-mediated membrane trafficking in podocytes. Although no significant changes were observed in microtubule modifications in cells expressing pathogenic INF2, subtle alterations in the levels, stability, modification, or distribution of microtubules cannot be ruled out. Therefore, using drugs that target microtubules may be beneficial for alleviating disease severity. The availability of INF2 R218Q KI mice and the development of protocols to induce FSGS in these models provide valuable opportunities to test such inhibitors in vivo.

## Future work

Significant progress has been made in understanding the regulation of INF2, yet several critical aspects remain unresolved. For instance, while the CAP/KAc-actin complex contributes to INF2 autoinhibition by facilitating the association of DID with the DAD, the regulation of the assembly and disassembly of the complex is still unclear. The cellular concentration of CAP proteins exceeds that of INF2, suggesting that the formation of complex is probably limited by the availability of KAc-actin, whose levels depend on the balance between KAT and KDAC. However, the specific enzymes involved and their regulation remain unidentified.

Additionally, it is also intriguing to explore whether coordination between Ca^2+^ signaling and KAc-actin deacetylation is required or whether the binding of Ca^2+^/CaM (centrin) to the N-terminal extension releases CAP/KAc-actin from INF2. The role of free G-actin in activating INF2 merits further investigation, as evidence shows that the G-actin/F-actin ratio is lower in INF2 KO cells than in control cells, and decreases in control cells upon activation of endogenous INF2. Further analysis is needed to determine whether Cdc42 has a direct role in regulating INF2 activity. Addressing all these questions could provide deeper insights into the molecular mechanisms governing INF2 regulation, potentially revealing new therapeutic targets for conditions linked to INF2 dysfunction.

Eighty INF2 variants are reported to be pathogenic, but this number could be significantly greater with comprehensive genetic analyses of unexplored DID mutants predicted to be pathogenic. While most pathogenic variants show similar deregulated actin polymerization activity in vitro [39, 48], the reasons behind the varying disease severities remain unclear, particularly why some lead solely to FSGS while others are associated with both FSGS and CMTD. Investigating the resistance mechanisms of nontransformed cells could provide insight into the role of INF2 in specific cancers and help explain the differential susceptibility of podocytes, Schwann cells, and sensory neurons, especially if the observed axonal loss is a direct effect. This research could also clarify why most cell types in the body remain affected.

Most of our knowledge about pathogenic INF2 has come from trying to explain INF2-linked FSGS, leaving the mechanisms leading to INF2-linked CMTD disease mostly unexplored. Electron microscopic examination of the distal nerves of patients revealed the presence of aberrant Schwann cells with an abnormally high content of F-actin in their cytoplasm [84, 107]. This finding is consistent with the constitutive activity of pathogenic INF2, which is likely to impair myelin formation and/or maintenance. Mitochondrial mobility defects have been suggested as a cause of INF2-linked CMTD [108].The development of KI mice that express pathogenic INF2 and that reproduce the symptoms of CMTD could provide a model for investigating the mechanisms underlying INF2-linked CMTD.

## Concluding remarks

The discovery of the linkage of INF2 to FSGS and subsequently to FSGS combined with CMTD, has sparked considerable interest in the study of INF2 function and regulation. This has also encouraged geneticists to search for novel INF2 DID variants that cause disease. This growing interest is reflected in more than 100 publications, nearly 70 of which report genetic analyses of pathogenic INF2 variants. Our in silico analysis suggests that the number of disease-causing INF2 DID mutations is much greater and that some WH2/DAD variants could be linked to disease.

Ca^2+^ signaling plays a crucial role in maintaining the morphology and architecture of podocytes, and its disruption leads to disease. For instance, autosomal dominant FSGS caused by mutation of the TRPC6 cation channel, as well as other podocyte-related diseases, including diabetic kidney disease, lupus nephritis, transplant glomerulopathy, and hypertensive renal injury, are associated with abnormal Ca^2+^ levels [109, 110]. Various biochemical processes and pathways, including mitochondrial fission and Ca^2+^-mediated activation of calcineurin and CaMK4 signaling, have been implicated in these diseases [109]. It is plausible that, in addition to monogenic diseases caused by pathogenic INF2 mutations, normal INF2 could be aberrantly activated in conditions with altered intracellular Ca^2+^ levels, contributing to renal disease.

Most mutations causing FSGS occur in genes related to the structure of slit diaphragm proteins that interact directly with the cytoskeleton or influence actin dynamics [111]. The importance of actin homeostasis is underscored by studies in podocyte-specific profilin1 KO mice, where podocytes undergo multinucleation and mitotic catastrophe [112]. Unlike loss-of-function, as occurs in profilin KO cells, pathogenic INF2 mutations cause FSGS through a gain-of-function mechanism, consistent with the Mendelian-dominant inheritance of INF2-linked disease.

Future research should focus on identifying the detailed mechanisms of INF2 regulation and its pathogenic variants, exploring potential therapeutic strategies, and understanding the broader implications of INF2 activity in glomerular and neurological diseases. This will not only deepen our knowledge of the role of INF2 in health and disease, but also pave the way for developing targeted treatments for INF2-linked conditions.

## Supplementary Information

Below is the link to the electronic supplementary material.

Supplementary file1 (PDF 421 KB)

Supplementary file2 (PDF 55 KB)

Supplementary file3 (XLSX 429 KB)

## Data Availability

Not applicable.
