# Supplementary material for: Regulation of formin INF2 and its alteration in INF2-linked inherited disorders
Source: Cell Mol Life Sci. 2024 Nov 25;81(1):463. doi: 10.1007/s00018-024-05499-3 (PMC11589041; doi:10.1007/s00018-024-05499-3)
Supplement: Supplementary file 1 — Supplementary file1 (PDF 421 KB) [file 18_2024_5499_MOESM1_ESM.pdf]

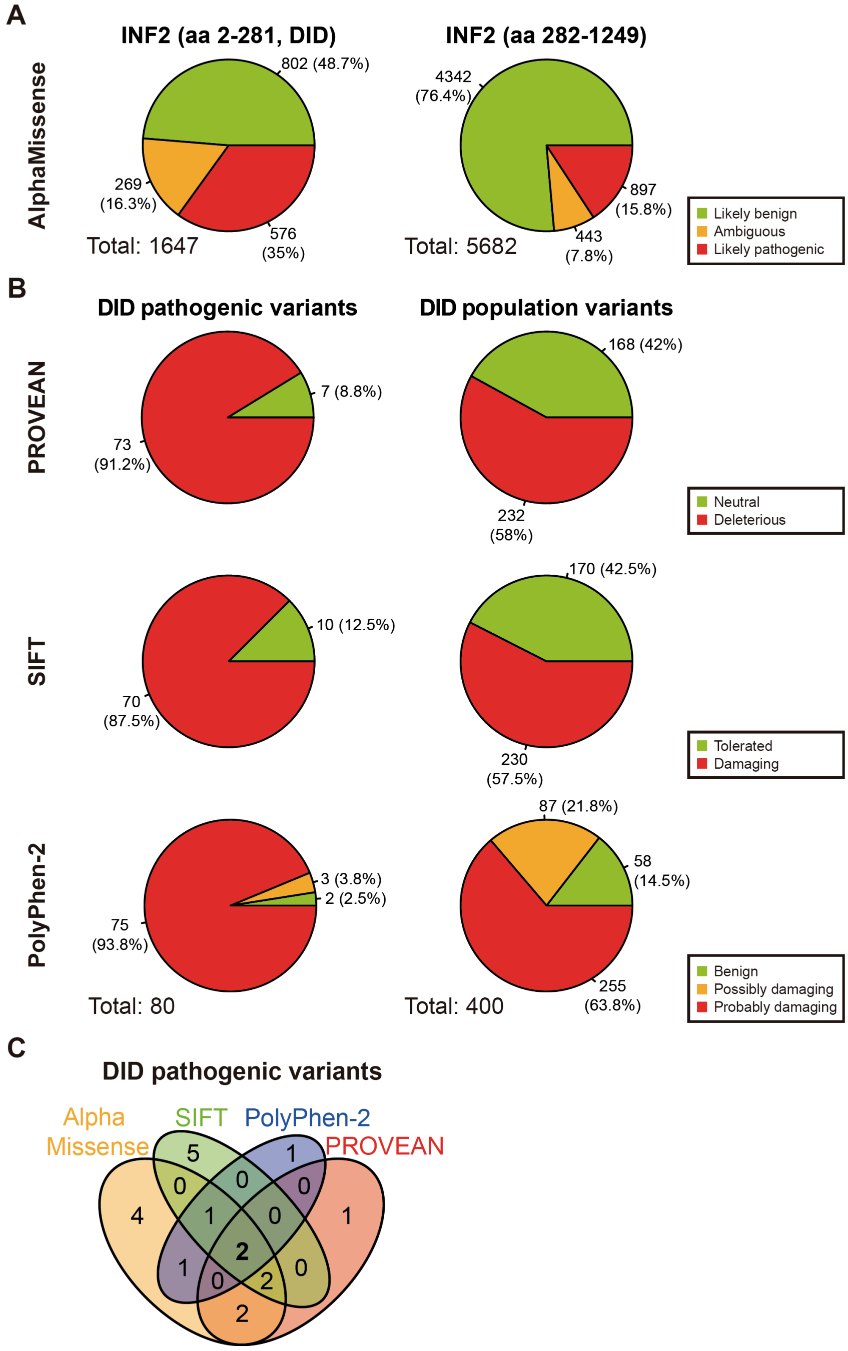

Fig S1 Pathogenicity predictions for INF2. (**A**) AlphaMissense was used to analyze the pathogenicity of missense mutants in two INF2 fragments: the 282-1249 fragment, which spans the entire INF2 sequence excluding the N-terminal extension and the DID, and the 2-281 fragment, which contains the N-terminal extension and the DID. (**B**) The pathogenicity of 80 missense variants in the 2-281 INF2 fragment reported to be pathogenic (left panels) and four hundred variants annotated in public databases (right panels) were analyzed with PROVEAN, SIFT and PolyPhen-2, as indicated. (**C**) Venn diagram showing the benignity predictions obtained from the AlphaMissense, PROVEAN, SIFT and PolyPhen-2 algorithms for 19 of the 80 human INF2 variants reported to be pathogenic but considered benign by at least one of the four algorithms. The matched predictions suggest that at least two of these variants are benign.
