# Supplementary material for: Regulation of formin INF2 and its alteration in INF2-linked inherited disorders
Source: Cell Mol Life Sci. 2024 Nov 25;81(1):463. doi: 10.1007/s00018-024-05499-3 (PMC11589041; doi:10.1007/s00018-024-05499-3)
Supplement: Supplementary file 2 — Supplementary file2 (PDF 55 KB) [file 18_2024_5499_MOESM2_ESM.pdf]

Table S1 Potential missense mutations resulting from single nucleotide substitutions in the INF2 1-281 fragment, which contains the DID. The table includes the mutations reported to be pathogenic, those found in the population, and all other possible missense mutations. Potential pathogenicity was analyzed using AlphaMissense, PROVEAN, SIFT and PolyPhen-2.

Table S2 Frequency of amino acid substitutions in the DID of INF2 in the pathogenic group of mutations. Amino acids are denoted using the single-letter code; aa1 indicates the residue in the wild type INF2 sequence and aa2 represent the residue in the variant.

Table S3 Potential missense mutations resulting from single nucleotide substitutions in the INF2-1 282-1249 fragment. Potential pathogenicity was analyzed using AlphaMissense.

Table S4 Missense mutations in the INF2 1-281 fragment found in the "population group", classified as likely pathogenic by the four predictors, and listed in ClinVar.

Table S5 Potential missense mutations resulting from single nucleotide substitutions in the INF2 967-991 fragment containing the DAD. Potential pathogenicity was analyzed using AlphaMissense, PROVEAN, SIFT and PolyPhen-2. The presence of the mutation in public databases and, specifically, in ClinVar is shown.
